# Diabetes Mellitus, Hypertension, and Death among 32 Patients with MERS-CoV Infection, Saudi Arabia

**DOI:** 10.3201/eid2601.190952

**Published:** 2020-01

**Authors:** Khalid H. Alanazi, Glen R. Abedi, Claire M. Midgley, Abdulrahim Alkhamis, Taghreed Alsaqer, Abdullah Almoaddi, Abdullah Algwizani, Sameeh S. Ghazal, Abdullah M. Assiri, Hani Jokhdar, Susan I. Gerber, Hail Alabdely, John T. Watson

**Affiliations:** Ministry of Health, Riyadh, Saudi Arabia (K.H. Alanazi, A. Alkhamis, T. Alsaqer, A. Almoaddi, A. Algwizani, S.S. Ghazal, A.M. Assiri, H. Jokhdar, H. Alabdely);; Centers for Disease Control and Prevention, Atlanta, Georgia, USA (G.R. Abedi, C.M. Midgley, S.I. Gerber, J.T. Watson)

**Keywords:** diabetes mellitus, hypertension, death, Middle East respiratory syndrome coronavirus, MERS-CoV, viruses, infection, vector-borne infections, zoonoses, underlying conditions, Saudi Arabia

## Abstract

Diabetes mellitus and hypertension are recognized risk factors for severe clinical outcomes, including death, associated with Middle East respiratory syndrome coronavirus infection. Among 32 virus-infected patients in Saudi Arabia, severity of illness and frequency of death corresponded closely with presence of multiple and more severe underlying conditions.

First described in 2012, infection with Middle East respiratory syndrome coronavirus (MERS-CoV) has been reported worldwide. More than 2,200 cases have been reported to the World Health Organization, and more than one third have resulted in death (*1*).

Certain underlying conditions, including diabetes mellitus (DM), hypertension, chronic cardiac disease, and chronic renal disease, are recognized risk factors for illness and death caused by infection with MERS-CoV (*2*,*3*). We further explored this relationship among MERS patients admitted to a referral hospital in Riyadh, Saudi Arabia, during August 1, 2015–August 31, 2016. Enrollment criteria and data collection methods have been described (*4*).

We considered persons with a medical history of DM as having documented DM and persons with multiple recorded periods of hyperglycemia during hospitalization as having possible DM (*4*). We similarly identified patients with hypertension or chronic kidney disease (CKD) by using documentation in the medical chart. We defined cardiovascular disease as having documentation of coronary artery disease or a history of heart failure or stroke. We considered patients with cardiovascular disease or CKD to have chronic organ damage (COD). We performed statistical analysis by using SAS version 9.4 (https://www.sas.com) and Microsoft Excel (https://www.microsoft.com).

Of 33 enrolled patients, medical history was available for 32 through medical charts. Underlying disease status among the 32 patients were no DM, hypertension, or COD (n = 11); DM without hypertension or COD (n = 5); DM and hypertension without COD (n = 5); and DM or hypertension with COD (n = 11). Of the 21 patients who had DM, 19 had DM documented in the medical chart, and 2 had possible DM, with random glucose readings >350 mg/dL during hospitalization. All 15 patients with hypertension had concomitant DM (Figure; Appendix Table). Of the 11 patients who had COD, 8 had cardiovascular disease, 1 had CKD, and 2 had cardiovascular disease and CKD. Of the 10 patients who had cardiovascular disease, 5 had a history of coronary disease, 3 had a history of heart failure, and 4 had a history of stroke; of the 3 patients with a history of heart failure, 1 also had coronary disease and 1 had a history of stroke.

**Figure:**
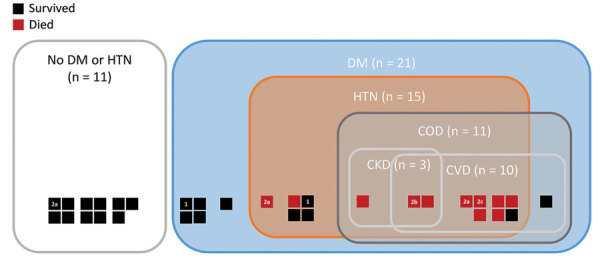
Characteristics of 32 case-patients infected with Middle East respiratory syndrome coronavirus, by underlying condition and survival status, Saudi Arabia. 1, DM defined as hyperglycemia recorded during hospitalization (n = 2). 2a, chronic lung disease in 1 patient with no DM or HTN who survived, 1 patient with DM and HTN but no COD who died, and 1 patient with CVD and not CKD who died. 2b, epilepsy in 1 patient with CVD and CKD who died. 2c, uterine cancer in 1 patient with CVD and not CKD who died. COD, chronic organ damage; CKD, chronic kidney disease; CVD, cardiovascular disease; DM, diabetes mellitus; HTN, hypertension.

Age was associated with presence of DM (mean 59 years vs. 30 years; p<0.0001, by *t*-test), hypertension (mean 64 years vs. 36 years; p<0.0001 by *t*-test), and cardiovascular disease (median 66 years vs. 41 years; p<0.0001 by *t*-test). Sex was not significantly associated with DM (68% of patients with DM were male vs. 45% of patients without DM; p = 0.2159 by χ^2^ test), hypertension (67% of patients with hypertension were male vs. 59% of patients without hypertension; p = 0.6474 by χ^2^ test), or cardiovascular disease (60% of patients with cardiovascular disease were male vs. 64% of patients without cardiovascular disease; p = 0.8439 by χ^2^ test).

Of the 32 patients, 21 survived until discharge, including 8 who required supplemental oxygen during hospitalization. Eleven died in the hospital, all of whom required ventilatory support. Case-patients with multiple and more severe underlying conditions generally had worse clinical course and outcomes than those without these conditions (Figure; Appendix Table). Of the 11 case-patients with no DM or hypertension, 100% survived, as did all patients with DM but without hypertension or COD. In comparison, 2 (40%) of 5 with DM and hypertension but without COD died, and 9 (82%) of 11 with DM or hypertension and with COD died (Appendix Table).

In a previous study (*5*), DM, chronic lung disease, heart disease, and smoking were identified as underlying health conditions and behaviors associated with primary infection with MERS-CoV. Alqahtani et al. reported that DM, hypertension, cardiac disease, renal disease, and bronchial asthma were frequent underlying conditions associated with death of MERS-CoV-infected patients and also found that the risk for death increased for patients with multiple comorbidities (*3*). We found that DM, hypertension, and COD co-occurred frequently in MERS-CoV–infected patients, and severity of illness and frequency of death were higher for patients with multiple and more severe underlying conditions. Further studies are necessary to better clarify the mechanisms that lead to severe outcomes among these patients.

For case-patients infected with MERS-CoV, the presence and compounding of underlying conditions, including DM, hypertension, and, ultimately, COD, corresponded with an increasingly complicated clinical course and death. These findings indicate that increased clinical vigilance is warranted for patients with multiple and severe underlying conditions who are suspected of being infected with MERS-CoV.

Appendix Additional information on diabetes mellitus, hypertension, and death among 32 patients with MERS-CoV infection, Saudi Arabia.
