# Supplementary material for: Diabetes Mellitus, Hypertension, and Death among 32 Patients with MERS-CoV Infection, Saudi Arabia
Source: Emerg Infect Dis. 2020 Jan;26(1):166–8. doi: 10.3201/eid2601.190952 (PMC6924889; doi:10.3201/eid2601.190952)
Supplement: Appendix — Additional information on diabetes mellitus, hypertension, and death among 32 patients with MERS-CoV infection, Saudi Arabia. [file 19-0952-Techapp-s1.pdf]

Article DOI: https://doi.org/10.3201/eid26011.19-0952

Diabetes Mellitus, Hypertension, and Death among 32 Patients with MERS-CoV Infection, Saudi Arabia

Appendix

Appendix Table. Underlying conditions and severity of outcome for 32 patients hospitalized with MERS-CoV infection, Saudi Arabia*

Characteristic	No DM, HTN, or COC	DM, no HTN or COD	DM and HTN, no COD	DM, HTN, and COD	Total
No. patients	11	5	5	11	32
No O_2 req., discharged	10 (77)	1 (8)	1 (8)	1 (8)	13
O_2 req., discharged	1 (13)	4 (50)	2 (25)	1 (13)	8
O_2 req., died	0	0	2 (18)	9 (82)	11

*Values are number or no. (%). COD, chronic organ disease; DM, diabetes mellitus; HTN, hypertension; MERS-CoV, Middle East respiratory syndrome coronavirus; O_2 req., supplemental oxygen requirement.
